# Effect of Asymptomatic Hyperuricemia on Mortality of Elderly Patients After Elective Percutaneous Coronary Intervention

**DOI:** 10.3389/fcvm.2022.800414

**Published:** 2022-03-17

**Authors:** Chen Chen, Jianzeng Dong, Qiang Lv, Xinmin Liu, Qian Zhang, Xin Du

**Affiliations:** Department of Cardiology, Beijing Anzhen Hospital, Capital Medical University, Beijing, China

**Keywords:** uric acid, prognosis, asymptomatic hyperuricemia, percutaneous coronary intervention (PCI), elderly

## Abstract

**Purpose:**

The purpose of this study is to investigate the effect of asymptomatic hyperuricemia on mortality of elderly patients with coronary artery disease (CAD) after elective percutaneous coronary intervention (PCI).

**Methods:**

One thousand two hundred ninety-six patients with coronary heart disease ≥65 years old who had increased uric acid records and without gout history underwent elective PCI from January 2015 to January 2016 were enrolled. The hyperuricemia is defined as serum uric acid level >420 μ mol/l (7 mg/dl) for males and >357 μ mol/l (6 mg/dl) for females. Patients were divided into hyperuricemia group and non-hyperuricemia group. After an average of 519 days follow-up, the differences in mortality between the two groups were compared.

**Results:**

There were 236 patients in hyperuricemia group and 1060 patients in non-hyperuricemia group. In hyperuricemia group, BMI was higher (*P* = 0.036); the proportions of patients with hypertension (*P* < 0.001) and myocardial infarction history (*P* = 0.046) were higher; white blood cells (*P* = 0.015) and triglyceride levels were higher (*P* < 0.001); and estimated glomerular filtration rate (*P* < 0.001) and high-density lipoprotein cholesterol level were lower (*P* = 0.007). In addition, in hyperuricemia group, during hospitalization, the ratios of patients treated with diuretics (*P* < 0.001) and the number of PCI lesions were higher (*P* = 0.030), and the complete revascularization rate was lower (*P* = 0.017). The mortality rate (2.2 vs. 7.6%, *P* < 0.001) of hyperuricemia group was significantly higher than that of non-hyperuricemia group. Multivariate Cox regression analysis showed that after adjusting for other factors, hyperuricemia was an independent risk factor for increased mortality after PCI (HR 2.786, 95% CI 1.233–6.297, *P* = 0.014).

**Conclusion:**

Asymptomatic hyperuricemia is an independent risk factor for increased mortality of elderly patients with coronary heart disease undergoing elective PCI.

## Introduction

At present, cardiovascular diseases have become the most common cause of death with coronary artery disease (CAD) taking up the largest proportion (1). Recent studies suggest that hyperuricemia is related to many cardiovascular risk factors, including age, sex, hypertension, diabetes, hypertriglyceridemia, obesity, insulin resistance, etc. (2–4). At present, aging has become an irreversible global trend. The number of elderly patients with CAD is increasing year by year. Percutaneous coronary intervention (PCI) has been widely used in the treatment of elderly patients with CAD. Accurate risk stratification and prognosis evaluation of elderly patients with CAD are extremely important for clinicians to identify high-risk individuals. At present, there are few studies on the effect of hyperuricemia on the prognosis of elderly patients with coronary heart disease after PCI. The purpose of this study is to investigate the effect of asymptomatic hyperuricemia on mortality of elderly patients with CAD undergoing elective PCI.

## Materials and Methods

### Study Group

This research retrospectively selected patients who received elective PCI treatment and had preoperative serum uric acid records without gout history from January 2015 to January 2016 in the Department of Cardiology, Beijing Anzhen Hospital. Indications of PCI were objective evidence of myocardial ischemia (positive sign of stress test) or ischemic symptoms associated with significant angiographic stenosis (stenosis > 75%). Elderly patients were defined as aged ≥ 65 years. Hyperuricemia is defined as serum uric acid level >420 μ mol/l (7 mg/dl) for males and > 357 μ mol/l (6 mg/dl) for females (5). Patients were divided into hyperuricemia group and non-hyperuricemia group according to their serum uric acid level. Data of cardiovascular risk factors, clinical features, laboratory tests, medications, angiographic and procedural information were collected in all patients.

### Data Source and Clinical Outcomes

The original data related to this research were collected from the standard medical records of the hospital by the researchers. The laboratory examinations and echocardiogram data were collected from admission to procedure. Among them, the laboratory tests were all fasting test results on the preoperative mornings, and the tests were carried out by the laboratories of hospital according to unified standards. All selected and follow-up data were conducted by cardiologists. Outpatient or telephone follow-ups were conducted according to a pre-designed questionnaire. Follow-up data were filled in a unified form and entered into the computer database. The endpoint of the research was all-cause death after PCI.

### Statistical Analysis

Continuous variables were expressed as mean and standard deviation. Continuous variables were compared by either Student's *t*-test or Mann-Whitney *U*-test. Categorical variables were analyzed using chi-square test or Fisher's exact test. Cox regression model was used for the analysis of the relevant factors affecting the mortality of the patients. Univariate analysis was performed with the following variables: hyperuricemia, age, gender, body mass index (BMI) at admission, hypertension, diabetes, previous stroke, previous myocardial infarction, current smoking, family history of CAD, previous PCI, diagnosis of acute ST-segment elevation myocardial infarction (STEMI) at admission, systolic blood pressure (SBP), left ventricular ejection fraction (LVEF), white blood cells, hemoglobin, estimated glomerular filtration rate (eGFR), total cholesterol, low density lipoprotein cholesterol (LDL-C), high density lipoprotein cholesterol (HDL-C), triglyceride, fasting blood glucose, medication during hospitalization, multivessel disease, left main lesion, proximal left anterior descending (LAD) lesion, chronic total occlusion (CTO), number of PCI vessels, number of PCI lesions and complete revascularization. Variables with *P* < 0.2 in univariate analysis were introduced into Cox regression for multivariate analysis. The hazard ratio (HR) and 95% confidence interval (95% CI) of all relevant factors were calculated through two-sided tests, with *P* < 0.05 showing statistical significance. All data were analyzed using statistical software SPSS 13.0.

## Results

### Baseline Data

In total, 1,296 patients were enrolled, including 889 males and 407 females. Mean age was 70 ± 4. There were 236 patients in hyperuricemia group and 1,060 patients in non-hyperuricemia group.

In hyperuricemia group, 22 cases were admitted with STEMI, 13 cases with NSTEMI (non-ST-segment elevation myocardial infarction), 164 cases with UAP (unstable angina pectoris), and 37 cases with SAP (stable angina pectoris). In non-hyperuricemia group, 96 cases were admitted with STEMI, 50 cases with NSTEMI, 705 cases with UAP, and 209 cases with SAP. In hyperuricemia group, BMI was higher (25.7 ± 3.7 vs. 25.1 ± 3.1, *P* = 0.036); the proportions of patients with hypertension (78.8 vs. 63.4%, *P* < 0.001) and myocardial infarction history (25.8 vs. 20.0%, *P* = 0.046) were higher; WBC (7.4 ± 1.9 vs. 7.1 ± 2.0 × 10^9^/L, *P* = 0.015) and TG levels were higher (1.8 ± 1.1 vs. 1.6 ± 0.9 mmol/L, *P* < 0.001); and eGFR (63 ± 23 vs. 81 ± 26 mL·min^−1^·1.73 m^−2^, *P* < 0.001) and HDL-C level were lower (1.02 ± 0.21 vs.1.07 ± 0.25 mmol/L, *P* = 0.007). In addition, in hyperuricemia group, the ratios of patients treated with diuretics (10.2 vs. 4.4%, *P* < 0.001) and the number of PCI lesions was higher (2.18 ± 1.32 vs. 1.96 ± 1.14, *P* = 0.030), and the complete revascularization rate was lower (76.3 vs. 82.9%, *P* = 0.017), as shown in Table 1.

**Table 1:** Baseline characteristics.

	**Non-hyperuricemia *n* = 1,060**	**Hyperuricemia** ***n* = 236**	***P* value**
Age, years, mean ± SD	70 ± 4	70 ± 4	0.252
Female, *n* (%)	323 (30.5)	84 (35.6.)	0.125
BMI, kg/m2, mean ± SD	25.1 ± 3.1	25.7 ± 3.7	0.036
Hypertension, *n* (%)	672 (63.4)	186 (78.8)	<0.001
Diabetes, *n* (%)	278 (26.2)	60 (25.4)	0.800
Previous stroke, *n* (%)	117 (11.0)	29 (12.3)	0.583
Previous myocardial infarction, *n* (%)	212 (20.0)	61 (25.8)	0.046
Current smoking, *n* (%)	324 (30.6)	72 (30.5)	0.986
Family history of CAD, *n* (%)	70 (6.6)	16 (6.8)	0.922
Previous PCI, *n* (%)	90 (8.5)	26 (11.0)	0.219
STEMI, *n* (%)	96 (9.1)	22 (9.3)	0.898
SBP, mmHg, mean ± SD	131 ± 19	132 ± 19	0.644
LVEF,%, mean ± SD	61 ± 11	60 ± 12	0.130
White blood cells,109/L, mean ± SD	7.1 ± 2.0	7.4 ± 1.9	0.015
Hemoglobin, g/L, mean ± SD	133 ± 16	133 ± 18	0.964
eGFR, mL min−1 1.73 m−2, mean ± SD	81 ± 26	63 ± 23	<0.001
Total cholesterol, mmol/L, mean ± SD	4.6 ± 1.1	4.8 ± 1.2	0.051
LDL-C, mmol/L, mean ± SD	2.7 ± 0.8	2.8 ± 0.7	0.351
HDL-C, mmol/L, mean ± SD	1.07 ± 0.25	1.02 ± 0.21	0.007
Triglyceride, mmol/L, mean ± SD	1.6 ± 0.9	1.8 ± 1.1	<0.001
Glucose mmol/L, mean ± SD	5.9 ± 1.8	5.8 ± 2.0	0.695
Medication, *n* (%)			
Beta blocker	947(89.3)	204(86.4)	0.201
Statins	778(73.4)	185(78.4)	0.112
ACEI or ARB	621 (58.6)	151 (64.0)	0.126
DAPT	1,019(96.1)	226(95.8)	0.792
Diuretics	47 (4.4)	24 (10.2)	<0.001
Procedural details, *n* (%)			
Multivessel disease	783(73.9)	180(76.3)	0.445
Left main lesion	115 (10.8)	28 (11.9)	0.653
Proximal LAD lesion	427 (40.3)	108 (45.8)	0.122
CTO lesion	206 (19.4)	53 (22.5)	0.293
Number of PCI vessels	1.50 ± 0.65	1.53 ± 0.67	0.684
Number of PCI lesions	1.96 ± 1.14	2.18 ± 1.32	0.030
Complete revascularization	879 (82.9)	180 (76.3)	0.017

*Data are presented as mean ± SD or n (%). BMI, body mass index; CAD, coronary artery disease; PCI, percutaneous coronary intervention; STEMI, ST segment elevation myocardial infarction; SBP, systolic blood pressure; LVEF, left ventricular ejection fraction; eGFR, estimated glomerular filtration rate; LDL-C, low density lipoprotein cholesterol; HDL-C, high density lipoprotein cholesterol; ACEI, angiotensin-converting enzyme inhibitor; ARB, angiotensin receptor blocker; DAPT, dual antiplatelet therapy; LAD, left anterior descending;CTO: chronic total occlusion*.

### Clinical Outcomes

**Figure 1:**
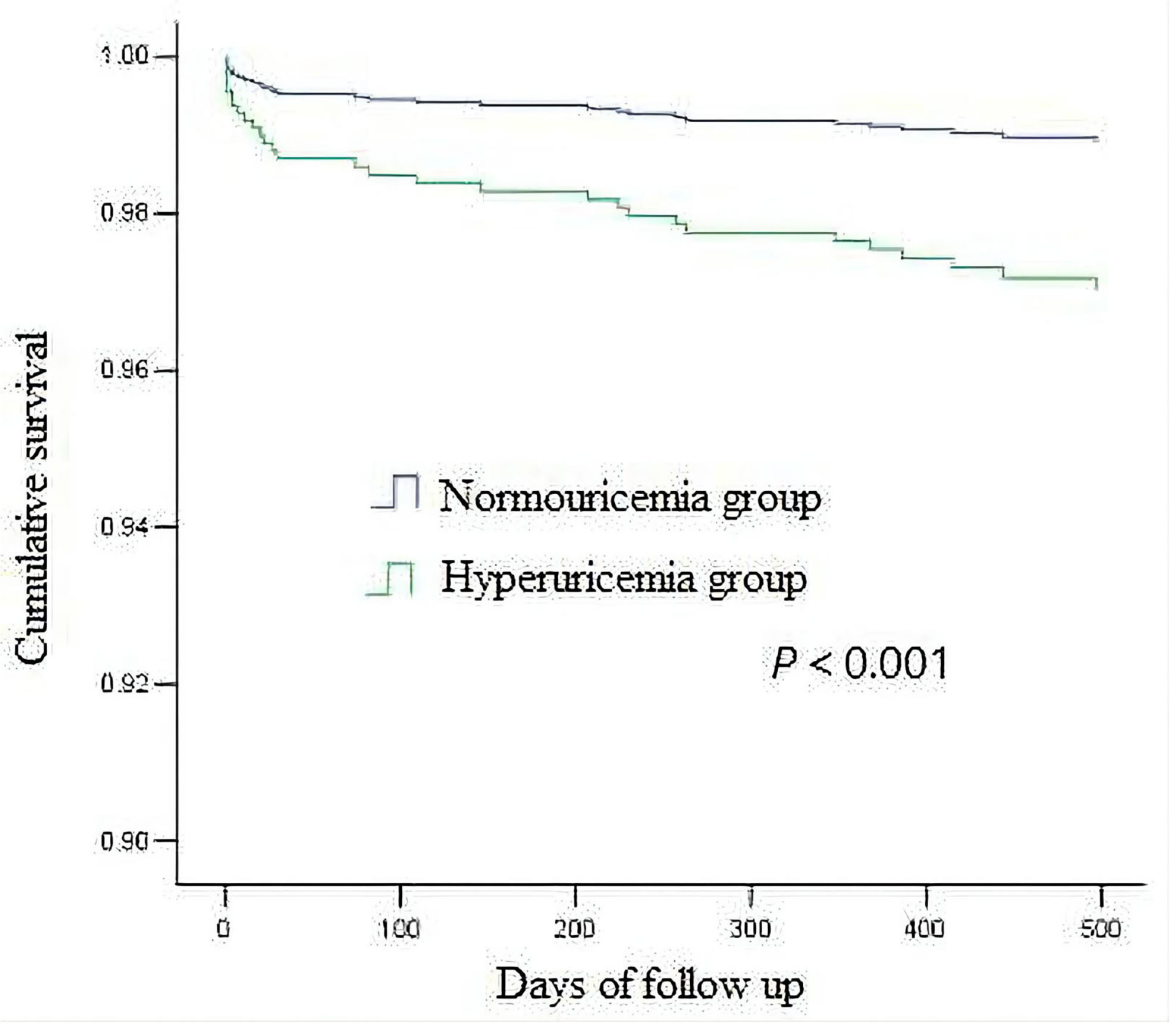
The Kaplan-Meier curve for cumulative survival.

The average follow-up was 519 days (325–713 days), and the follow-up rate was 94.7%. There were 18 postoperative deaths (7.6%) in hyperuricemia group and 23 postoperative deaths (2.2%) in non-hyperuricemia group, the difference was statistically significant (*P* < 0.001). Among them, 13 patients in non-hyperuricemia group died in hospital (1.2%), and 8 patients in hyperuricemia group died in hospital (3.4%), the difference was statistically significant (*P* = 0.039). In the univariate analysis of factors affecting mortality, the variables with *P* < 0.2 included hyperuricemia, age, BMI at admission, family history of CAD, previous PCI, LVEF, hemoglobin, eGFR, total cholesterol, medication (beta-blocker, statins, dual antiplatelet therapy (DAPT)), left main lesion, complete revascularization, as shown in Table 2. The above variables were introduced into Cox regression for multivariate analysis which showed that hyperuricemia (HR 2.786, 95% CI 1.233–6.297, *P* = 0.014), age (HR 1.177, 95% CI 1.089–1.274, *P* < 0.001),BMI (HR 0.874, 95% CI 0.769–0.993, *P* = 0.039), eGFR(HR 0.979, 95% CI 0.960–0.999, *P* = 0.035) before PCI and complete revascularization (HR 0.360, 95% CI 0.152–0.855, *P* = 0.021) were independent risk factors for postoperative death, as shown in Table 3. Kaplan-Meier survival curve showed that the survival rate of patients with high uric acid was lower than of those of normal uric acid (*P* < 0.001), as shown in Figure 1.

**Table 2:** Univariate analysis for all-cause mortality.

	**HR**	**95%CI**	***P* value**
Hyperuricemia	2.448	1.334–4.491	0.004
Age	1.139	1.071–1.211	<0.001
Female	1.154	0.629–2.116	0.644
BMI	0.884	0.795–0.983	0.023
Hypertension	1.265	0.707–2.263	0.429
Diabetes	1.197	0.688–2.085	0.524
Previous stroke	1.362	0.609–3.046	0.451
Previous myocardial infarction	1.181	0.600–2.325	0.631
Current smoking	1.051	0.729–1.541	0.790
Family history of CAD	1.696	1.030–2.790	0.038
Previous PCI	0.224	0.031–1.629	0.140
STEMI	0.948	0.340–2.644	0.919
SBP	0.998	0.983–1.013	0.769
LVEF	0.972	0.951–0.994	0.011
White blood cells	1.039	0.906–1.192	0.582
Hemoglobin	0.984	0.967–1.001	0.062
eGFR (ml · min−1 · 1.73 m−2)	0.968	0.953–0.984	<0.001
Total cholesterol	1.005	0.999–1.012	0.101
LDL-C	1.005	0.996–1.013	0.315
HDL-C	0.983	0.952–1.017	0.295
Triglyceride	1.001	0.999–1.004	0.342
Glucose	1.004	0.998–1.012	0.209
Beta blocker	0.564	0.263–1.208	0.141
Statins	0.435	0.243–0.779	0.005
ACEI/ARB	1.027	0.568–1.856	0.931
DAPT	0.378	0.211–0.678	<0.001
Diuretics	0.595	0.081–4.375	0.610
Multivessel disease	1.643	0.766–3.521	0.202
Left main lesion	1.957	0.944–4.054	0.071
Proximal LAD lesion	1.427	0.801–2.543	0.228
CTO lesion	0.985	0.476–2.042	0.968
Number of PCI vessels	1.232	0.656–2.314	0.517
Number of PCI lesions	0.805	0.520–1.246	0.331
Complete revascularization	0.408	0.224–0748	0.004

*BMI, body mass index; CAD, coronary artery disease; PCI, percutaneous coronary intervention; STEMI, ST segment elevation myocardial infarction; SBP, systolic blood pressure; LVEF, left ventricular ejection fraction; eGFR, estimated glomerular filtration rate; LDL-C, low density lipoprotein cholesterol; HDL-C, high density lipoprotein cholesterol; ACEI, angiotensin-converting enzyme inhibitor; ARB, angiotensin receptor blocker; DAPT, dual antiplatelet therapy; LAD, left anterior descending; CTO, chronic total occlusion*.

**Table 3:** Multivariate analysis for all-cause mortality.

	**HR**	**95%CI**	***P* value**
Hyperuricemia	2.786	1.233~6.297	0.014
Age	1.177	1.089~1.274	<0.001
BMI	0.874	0.769~0.993	0.039
eGFR	0.979	0.960~0.999	0.035
Complete revascularization	0.360	0.152~0.855	0.021

*BMI, body mass index; eGFR, estimated glomerular filtration rate*.

## Discussion

Uric acid is a product of purine metabolism in human body, and the increase of uric acid level is related to abnormal nucleic acid metabolism *in vivo* and reduction of renal excretion. The diagnostic criteria for hyperuricemia is defined as serum uric acid level > 420 μ mol/L (7 mg/dl) for males and > 357 μ mol/L (6 mg/dl) for females. Hyperuricemia (HUA) without gout attack is called asymptomatic hyperuricemia (6).

Baseline data of this study showed that in hyperuricemia group, BMI was higher; the proportions of patients with hypertension and previous myocardial infarction were higher; WBC and TG levels were higher; eGFR and HDL-C level were lower. In addition, in hyperuricemia group, during hospitalization, the ratios treated with diuretics and the number of PCI lesions was higher; and the complete revascularization rate was lower. After adjusted by relevant risk factors, hyperuricemia is still an independent risk factor for increased post-PCI mortality in elderly patients with CAD.

In recent years, studies have shown that hyperuricemia is a risk factor for cardiovascular diseases and is closely related to the occurrence and development of cardiovascular diseases. Serum uric acid is an indicator that is easy to monitor and can be treated in daily clinical practice. Recent studies on the involvement of hyperuricemia in atherosclerosis demonstrated that treatment with xanthine oxidase inhibitors not only facilitates the treatment of hyperuricemia, but also reduces the burden of atherosclerosis to a great extent (5). It is of great significance to intervene this risk factor and improve the prognosis of patients.

Research by Bickel et al. showed that (7), patients with CAD confirmed by coronary angiography were followed up for an average of 2.2 years. Patients with serum uric acid level in the highest quartile (>7.1 mg/gl), the mortality rate was 17.1%, and for patients with serum uric acid level in the lowest quartile (<5.1 mg/gl), the mortality rate was 3.4%. After adjusting for age, gender and other factors, the mortality rate of patients with CAD increased as their serum uric acid level increased, with the female HR being 1.30 (95%CI 1.14–1.49, *P* ≤ 0.001) and the male HR being 1.39 (95%CI 1.21–1.59, *P* ≤ 0.001). Multivariate regression analysis showed that the increase of serum uric acid level was independently and significantly correlated with the mortality rate of patients with CAD. Clinical research showed that hyperuricemia can increase the post-PCI mortality. The research of Spoon et al. (8) included 1916 patients with uric acid records within 2 years before PCI. Diagnostic criteria for hyperuricemia were defined as male uric acid >8 mg/dl and female uric acid > 7 mg/dl. The patients were divided into two groups with 1,353 patients in normal uric acid group and 563 patients in high uric acid group. After 2 years of follow-up, the mortality rate of patients in high uric acid group increased significantly (HR 1.25, 95%CI 0.98–1.59, *P* = 0.07). Tscharre et al. believed (9) that among patients with acute coronary syndrome undergoing PCI, hyperuricemia (female UA>6.0 mg/dL, male > 7.0 mg/dL) was independently associated with long-term major adverse cardiovascular events (MACE), including cardiovascular death, myocardial infarction and stroke. The cardiovascular mortality rate of patients with hyperuricemia increased by 1.6 times (*P* = 0.005), and the risk of myocardial infarction increased by 1.5 times (*P* = 0.032). A study from Mexico found that patients with asymptomatic hyperuricaemia had a higher proportion of complex coronary lesions and a higher SYNTAX I score (10).

The exact mechanism of hyperuricemia leading to poor prognosis of PCI is not clear, and it may function in the following ways: for patients with hyperuricemia, urate crystals in their blood are easy to deposit on arterial wall, damaging the intima and endothelial function of blood vessels. Uric acid can inhibit the production of nitric oxide, causing endothelium-dependent vasodilation dysfunction and vessel wall damage. High uric acid leads to increased production of oxygen free radicals and inflammatory factors, promotes platelet aggregation, contributes to arteriosclerosis and thrombosis, and speeds up vascular injury. Uric acid enhanced coronary atherosclerosis, reduces coronary blood flow reserve, and impairs coronary microvascular function (11–20).

In recent years, with the popularization of PCI, the number of patients receiving PCI has increased significantly. It is of great significance to identify new risk factors in these patients that are easy to monitor and can be treated, and then intervene these risk factors to improve the prognosis of patients.

And importance should be specially attached to the control of risk factors when patients with coronary heart disease are receiving elective PCI treatments, owing to the fact that they are usually in poor physical conditions and often threatened by multiple complications. Patients with high uric acid having no gout attacks tend to neglect the control of uric acid levels. Previous studies were not conducted on the prognosis of asymptomatic hyperuricemia in elderly patients with coronary heart disease who had received elective PCI treatment. The patients in this study, which constituted a large sample size, were consecutively included, thus being capable of reflecting the fact. The results of the study show that, among those elderly patients having no gout attacks who had received elective PCI treatment, 18.2% of them showed elevated uric acid. This is an indication of a huge number of the patients with asymptomatic hyperuricemia. What's worse is that, poor prognosis and high mortality were found in these patients. Therefore, it is of great significance to improve the prognosis of these patients. The results of this study further illustrate the importance of controlling uric acid levels in patients undergoing PCI treatment, which deserves the attention of clinicians.

The study also has some limitations. This was a single center, retrospective, exploratory study. Nowadays, with the improvement of PCI procedures, the postoperative and in-hospital mortality rates are becoming lower, only a small percentage patients occurred endpoint-events in this study, residual confounding factors possibly affected the results, the accuracy is still preliminary. Another limitation is that only intermediate-term follow-up results are available. Large-scale multicenter long-term follow-up trial is required to further verify the findings.

## Conclusion

In conclusion, the present study demonstrated that hyperuricemia is a predictive factor for all-cause mortality in elderly CAD patients after elective PCI. Hyperuricemia had the potential to become a new indicator in prognostic evaluation and risk stratification of the elderly patients with CAD.

## Data Availability Statement

The original contributions presented in the study are included in the article/supplementary material, further inquiries can be directed to the corresponding author.

## Ethics Statement

The studies involving human participants were reviewed and approved by the Ethics Committee on Human Research in Beijing Anzhen Hospital, the Capital Medical University. The patients/participants provided their written informed consent to participate in this study.

## Author Contributions

JD, XD, QL, and CC contributed to conception and design of the study. QL, XL, and CC collected data and organized the database. XL and QZ performed the statistical analysis. CC wrote the first draft of the manuscript. All authors contributed to manuscript revision, read, and approved the submitted version.

## Conflict of Interest

The authors declare that the research was conducted in the absence of any commercial or financial relationships that could be construed as a potential conflict of interest.

## Publisher's Note

All claims expressed in this article are solely those of the authors and do not necessarily represent those of their affiliated organizations, or those of the publisher, the editors and the reviewers. Any product that may be evaluated in this article, or claim that may be made by its manufacturer, is not guaranteed or endorsed by the publisher.
